# Presentation of a minimally symptomatic large extradural hematoma in a patient with an arachnoid cyst: a case report and review of the literature

**DOI:** 10.1186/1752-1947-5-581

**Published:** 2011-12-19

**Authors:** Afsoun Seddighi, Amir Saied Seddighi, Ali Reza Zali, Hesam Rahimi Baqdashti

**Affiliations:** 1 Department of Neurosurgery, Shahid Rajaee Hospital, Qazvin University of Medical Sciences, Tehran, Iran; 2 Functional Neurosurgery Research Center of Shohada Tajrish Hospital, Shahid Beheshti University of Medical Sciences, Tehran, Iran; 3 Department of Neurosurgery, Shohada Tajrish Hospital, Shahid Beheshti University of Medical Sciences, Tehran, Iran

## Abstract

**Introduction:**

Hemorrhagic complications of arachnoid cysts have been reported, extensively presenting with subdural or intracystic hematoma after trauma, but presentation with extradural hemorrhage is very rare. In this paper, we report the case of a patient with an arachnoid cyst who developed an extradural hematoma after a subtle head injury. Our patient presented with very mild symptoms in spite of the very large size of the hematoma.

**Case presentation:**

Our patient was a 23-year-old Iranian man who complained of diffuse progressive headache and vomiting after mild head trauma. A brain computerized scan showed a very large extradural hematoma in the left frontotemporoparietal convexity over a large arachnoid cyst.

**Conclusion:**

Brain parenchyma containing an arachnoid cyst is vulnerable to trauma and increases the risk of serious hemorrhagic complications. We also suggest that the abnormal shape of the head should be considered as an indication for a computerized tomography scan in cases of mild head injury.

## Introduction

Traumatic extradural hematomas (EDH) occur in approximately 1% to 5% of all cases of head injuries [1]. It has been regarded as an emergency and if not treated on time can cause rapid and lethal neurological complications. Therefore early diagnosis is extremely important for adequate patient management. However, in many of the reported series, many patients may present with subtle symptoms, such as headache, vomiting, vertigo and others, which may be neglected in the first examinations, especially in the setting of brain atrophy or anomalies [2,3].

There are many reports of arachnoid cysts in the literature presenting with subdural or intracystic hematoma following trauma [4-8], but presentation with extradural hemorrhage has extremely rarely been reported [9,10].

In this paper we report a patient with a very large traumatic epidural hematoma in the setting of an arachnoid cyst. In a review of the literature we found only six reported cases with the same diagnosis. An important feature of our patient was that he presented with very mild symptoms in spite of the huge size of the extradural hematoma.

## Case presentation

Our patient was a 23-year-old Iranian man who complained of diffuse headache and several bursts of vomiting on the day of presentation. He told us that three days before, he had received a traumatic head injury after falling from his bicycle at a low speed due to an obstacle. He had no post-traumatic amnesia, loss of consciousness, seizure or visual blurring. Initially, his headache was mild and focal, but its severity gradually increased. He had been visited by a general practitioner and a skull X-ray had been performed, which did not show any fracture line. The general practitioner had prescribed an acetaminophen tablet every eight hours. After two days, despite the medication, the severity of the headache had increased, become generalized and was not responding to the analgesic. The quality of headache was pulsatile without remission. He also developed nausea and vomiting.

Our patient had no history of seizure. He was uneducated but could cope with his daily life activities alone. He had been married for four years and had a two-year-old son. In a physical examination, macrocrania and asymmetry of his calvarium were evident, with a marked expansion of the left hemicranium. The deformity had been present for many years and our patient had been told that the bulging of his head was due to abnormal fluid accumulation; no neuroradiological investigation had been performed during his childhood.

**Figure 1:**
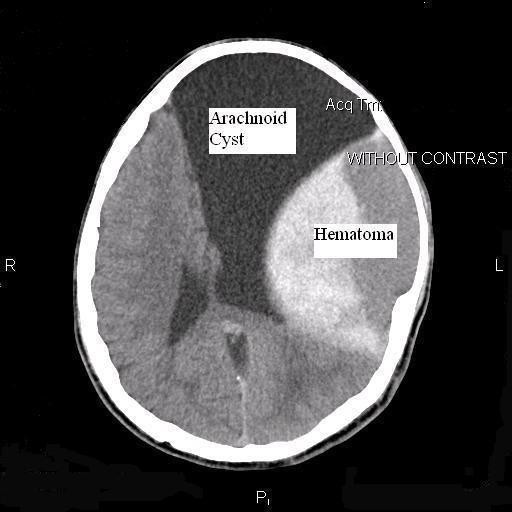
**Brain CT scan of our patient**. Shows the very large arachnoid cyst and extradural hematoma over the left frontoparietal convexity, with the bulged skull over the cyst.

In a neurological examination, his score on the Glasgow coma scale was 15 and his mental status was normal. In an ophthalmologic exam, he showed grade 2 papilledema. His pupils were normal size and the movements of his eyes were normal bilaterally. Motor and sensory exams were normal and his deep tendon reflexes were normal and symmetric bilaterally. A computerized tomography scan (CT scan) of his brain was performed, which revealed a very large extradural hematoma in the left frontotemporoparietal convexity, but surprisingly we also saw a very large cystic area over the frontoparietal convexity that extended into the left middle fossa. The overlying skull was bulged. The lesion appeared to be similar to an arachnoid cyst and was associated with the lentiform extradural hematoma, measuring 5.5 cm×7 cm, extending from the base of the middle cranial fossa to the middle part of the left parietal lobe with a total height of 6 cm. There was no evidence of a skull fracture and there was a left-to-right midline shift and the ipsilateral ventricle was compressed (Figure 1). Routine laboratory tests, including a complete blood cells count, coagulative profile and serum electrolytes, were within normal limits. Since our patient showed symptoms of increased intracranial pressure we decided to proceed with surgery.

We performed a left frontoparietal craniotomy. No skull fracture was detected. We then evacuated the extradural hematoma, which was similar in appearance to motor oil liquid. The source of the hematoma was a tear in the middle meningeal vein, which was coagulated with a bipolar cautery. We opened the dura with a small incision and the use of a microscope. The arachnoidal cyst was identified by the marked atrophy of the underlying brain. We did not find any subdural hematoma or intracystic hemorrhage.

**Figure 2:**
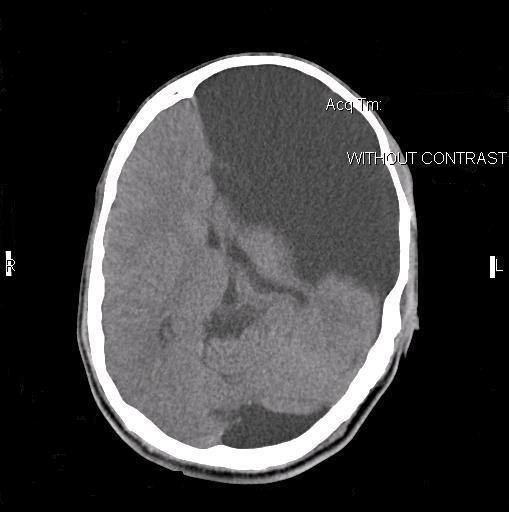
**Postoperative brain CT scan of our patient after evacuation of the hematoma**. Shows decompression of the cyst and underlying brain parenchyma.

**Figure 3:**
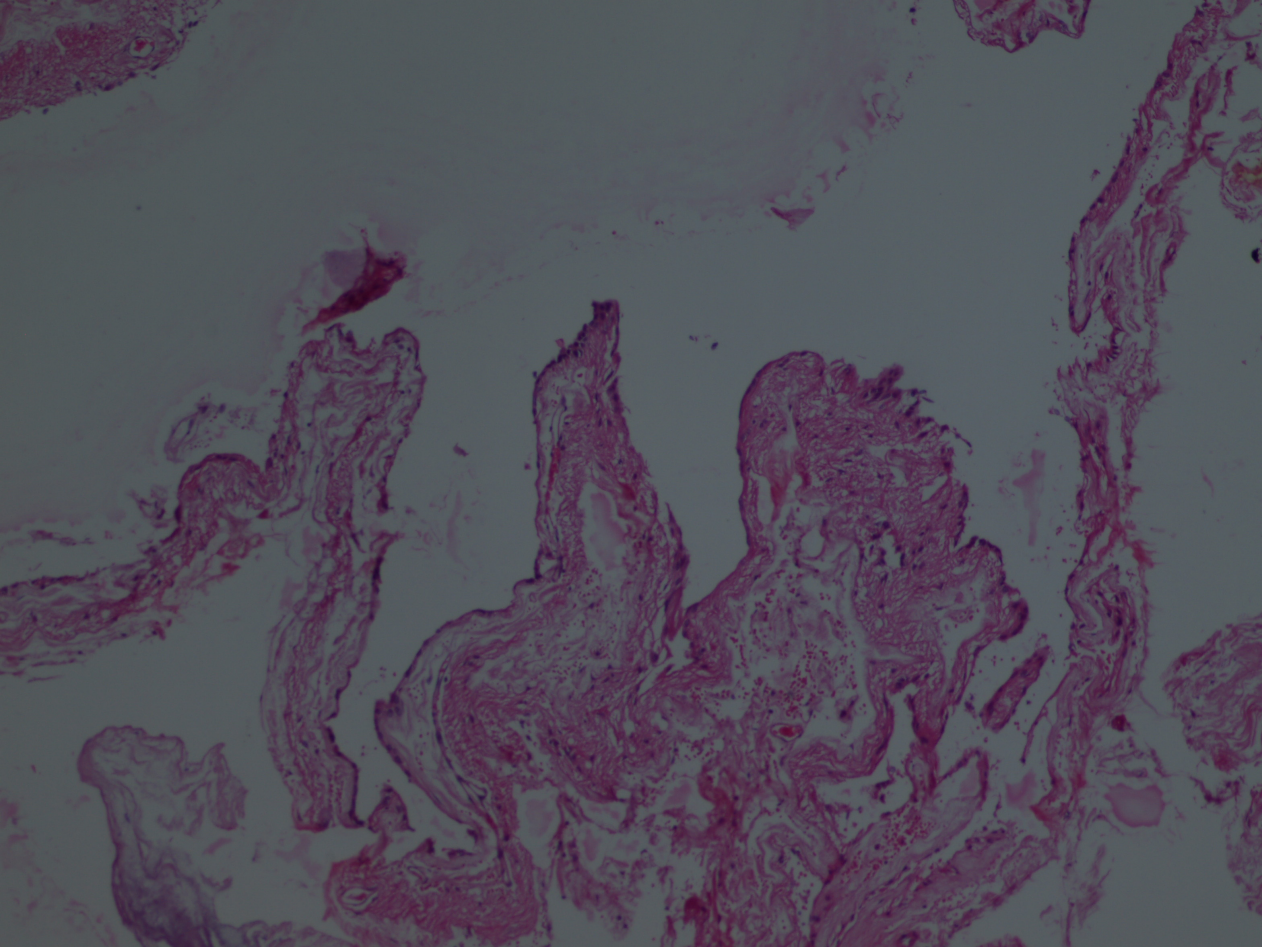
**Pathological specimen of the cyst wall**. Shows cyst and arachnoidal cells.

We decompressed the cyst and took a biopsy of the cyst membrane. After suspension of the dura with tack-up sutures, the wound was closed in layers. Our patient's postoperative course was excellent and uneventful. A postoperative brain CT scan of our patient showed evacuation of the hematoma and decompression of the cyst and underlying brain parenchyma (Figure 2). The pathologist reported the specimen to be arachnoid membrane (Figure 3). In the last follow-up visit, six month after surgery, our patient was symptom free, the papilledema was resolved, and he was working in a fast food kiosk.

## Discussion

Arachnoid cysts are congenital fluid-filled cavities, circumscribed by arachnoidal membrane, that have settled in the cisternae and major cerebral fissures. They constitute 1% of non-traumatic intracranial mass lesions [11]. Intracystic fluid resembles cerebrospinal fluid (CSF) [12]. When symptomatic, they usually present with symptoms of increased intracranial pressure. On the other hand, symptomatic children may present with an abnormal head shape, seizures and focal neurological deficits.

These lesions may be tolerated for a long time in life and may be diagnosed for the first time in the elderly, or may be an incidental finding [10,13]. With the advent of neuroimaging, there has been an increased incidence of detection of incidental asymptomatic arachnoid cysts. Hemorrhage into an arachnoid cyst and the associated subdural hematoma following head trauma are well documented, although the mechanism and true incidence are not clearly understood. The annual risk for hemorrhage in patients with a middle cranial fossa cyst probably remains below 0.1% [14].

Most authors agree that arachnoid cysts, although benign and often asymptomatic [10], are potential sources of complications, such as enlargement, subdural hygromas, subdural hematoma and hemorrhage within the cyst [10,15].

Using several search engines and databases, such as Google, PubMed, Ovid and AltaVista, we found only six cases of extradural hemorrhage in the context of arachnoid cyst. The first case was reported by Galassi in 1986 [10] and the most recent by Boviatsis in 2003 [9].

All of the reported cases were male patients [9,10,16-18], as in our case. In all of the reported cases, the arachnoid cyst occupied the Sylvian fissure. Sylvian arachnoid cysts are more common in men [19,20], and men are more prone to trauma, so this male predominant presentation could be attributed to these facts. Of course, this sex predilection could not be generalized, due to the small number of the reported cases.

Our patient was 23 years old. All of the other cases, except the patient reported by Boviatsis [9], were under 40. This can be attributed to the fact that arachnoid cysts are more prevalent in young age and trauma is also more prevalent in this age group [9].

In our case, like all the other patients, an epidural hematoma occurred ipsilateral to the arachnoid cyst [9,10,16-18]. The reported post-traumatic hemorrhagic complications in a setting of a temporal fossa arachnoid cyst were often confined to the same side as the cyst. This finding reinforces the fact that an arachnoid cyst, being a large fluid-filled lesion, is less compliant than normal brain parenchyma, making the ipsilateral side more prone to the formation of a hematoma [19,20].

In all of the reported cases, including ours, the hemorrhagic lesions were supratentorial, which may be explained by the fact that arachnoid cysts and head injuries are both more common in the supratentorial compartment [9,10,16-20].

Our patient had not suffered a skull fracture because he suffered from only a subtle head impact. In all of the other cases the head injury was of mild degree and no linear fracture was detected [9,10,16-18], except in one case reported by Galassi, where the patient experienced severe head trauma due to a high-velocity road traffic accident. Unlike the other cases, his patient presented with a coma and his brain CT scan showed a skull fracture [10]. According to the literature on large scale studies, the overall incidence of skull fracture ranges between 60% to 85% in traumatic epidural hematoma [21,22].

In cases with traumatic extradural hematoma, the major source of bleeding has been reported to be the rupture of the middle meningeal artery. Khan and Nadeem, in a review of a large series of traumatic cases with extradural hematoma, studied the source of bleeding and reported it to be a tear of the middle meningeal artery in 54% of cases, a rupture of the middle meningeal vein in nearly 12% of cases, a tear in the dural sinus in 13.5%, skull fracture and a tear in the diploic veins in 12.5% of cases, with no identifiable source in 8% of the cases [23].

An important feature is that the extradural hemorrhage in our patient and other reported cases were all of venous origin. The low-pressure blood extravasations had led to the development of the hematoma in the space previously occupied by the cyst. This would explain the quiet and slow clinical presentation in spite of very large size of the lesion [9,10,16-18].

The density of the hematoma in our patient was mixed, with hyperdense and hypodense components relative to the brain parenchyma. Extradural hematomas can have different densities related to the interval between the trauma and performance of the CT scan [24].

Acute, or type 1, EDH may contain both a hyperattenuating clot and a swirling lucency. These findings are believed to represent a mixture of active bleeding and the serum remaining after previous clot formation. Subacute, or type 2, EDH becomes homogeneously hyperattenuating as active bleeding ceases and an organized clot forms. Chronic, or type 3, EDH is at least partly hypoattenuating as the clot undergoes breakdown and absorption. The hematoma in our patient was partly hypodense and according to the above classification can be classified as type 3 or chronic [24], which can be related to its slow progression and the delay in diagnosis.

After we performed the craniotomy, the chronic extradural hematoma had the appearance of motor oil liquid and there was no CSF leak and capsule formation. This is similar to the description of chronic EDH (CEDH) by Merih [25]. Bradley defined CEDH as cases diagnosed more than 14 days after the head injury, based on the breakdown of hemoglobin products on T1- and T2- weighted MRI [26]. In our case, CEDH was diagnosed 72 hours after the head trauma. This time period is consistent with that found in a study by Sparacio *et al*. [27]. The period between the onset of injury and the time of the CT scan may vary considerably. The definition of CEDH is different according to different authors. Sparacio *et al*. [27] defined a CEDH as occurring 48 hours to 72 hours after trauma, whereas Iwakuma and Brunngraber [28] noted a 13-day interval for chronic EDH. It seems that there is not a clear cut distinction between acute and chronic lesions [29].

In a review of the literature we searched the indications for a CT scan in head trauma patients, but using several search engines and databases, such as Google, Yahoo!, PubMed and AltaVista, the shape of the head was not considered in these papers [24-30].

## Conclusion

It is of critical importance that EDHs be diagnosed as quickly as possible, because they are life threatening. The operative mortality is related directly to the level of consciousness at the time of surgery [29,30]. This study emphasizes that, in patients with traumatic head injury, the presence of worsening headache should never be underestimated and always requires more evaluation to exclude a slow progressive intracranial lesion, especially in the presence of an intracranial malformation. Although general conclusions cannot be based upon these limited observations, our report may represent further confirmation that a intracranial compartment containing an arachnoid cyst is vulnerable, and exposes the patient to the risk of serious hemorrhagic complications in case of trauma. Hemorrhagic complications of arachnoid cysts, with or without preceding cranial trauma, may be explained by many mechanisms, such as vascular conditions, membranous adhesions, diminished compliance and cushioning derived from masses with different physical properties [10,30].

We should inform patients with arachnoid cysts about the possibility of such complications accompanying a head injury in daily life. We also advocate that the abnormal shape and size of the head should be considered an indication for appropriate neuroimaging in patients with a mild head injury, even with subtle symptoms, in emergency units.

## Consent

Written informed consent was obtained from the patient for publication of this case report and any accompanying images. A copy of the written consent is available for review by the Editor-in-Chief of this journal.

## Abbreviations

CEDH: chronic epidural hematoma; CSF: cerebrospinal fluid; CT: computerized tomography; EDH: epidural hematoma: MRI: magnetic resonance imaging.

## Competing interests

The authors declare that they have no competing interests.

## Authors' contributions

AS analyzed and interpreted the patient data regarding the extradural hematoma in the setting of an arachnoid cyst and performed the surgery. ASS aided in the surgery, performed the literature review and was a major contributor in writing the manuscript. ARZ helped in the literature review and interpretation of the neuroimaging. HRB helped in the literature review and organized the references and figures. All authors read and approved the final manuscript.
